# How Can We Educate the Public?

**Published:** 1897-07

**Authors:** B. J. De Vries

**Affiliations:** Holland, Mich.


ARTICLE III.

### HOW CAN WE EDUCATE THE PUBLIC?

BY DR. B. J. DE VRIES, HOLLAND, MICH.

How shall we educate the public? The dental profession itself is inadequate to the task. Where better can we go for assistance than to that grand educator itself—the public schools? Already there has been added to the curriculum of our schools such branches as hygiene and physiology. Why not also give instruction in the care of the teeth? If one hour in every week was judiciously devoted to this subject it would be time well spent.

For instance, from charts with a few illustrations.

Give the number and names of the deciduous and permanent teeth; time of eruption; what kind of food is beneficial to tooth structure?

How should teeth be brushed? With what? etc.

I believe if some wide-awake dentist would write a small, attractive work on this subject, to be used in the public schools, he would confer a great benefit on society, and I am sure it would not be without remuneration. Society must learn that a dentist cannot save teeth without the assistance of their own efforts. There are those who will not, among men and women as well as children, but let us do something for those that know not how and why, who never come near our offices, and who are waiting to be taught that they may care for their own teeth as far as in their power lies, and to be able to discern when they need the assistance of a dentist.

We take it, then, for granted, that if we expect to see good results from our labors we must establish habits of cleanliness. The use of the brush and a proper antiseptic mouth-wash are essential parts to be performed by our young patients. And without these habits our work will be void.—*Register.*

